# Knee sensorimotor control following anterior cruciate ligament reconstruction: A comparison between reconstruction techniques

**DOI:** 10.1371/journal.pone.0205658

**Published:** 2018-11-15

**Authors:** Cristóbal San Martín-Mohr, Iver Cristi-Sánchez, Patricio A. Pincheira, Alvaro Reyes, Francisco José Berral, Claudio Oyarzo

**Affiliations:** 1 Carrera de Kinesiología, Departamento Ciencias de la salud, Facultad de Medicina, Pontificia Universidad Católica de Chile, Santiago, Chile; 2 Clínica MEDS, Santiago, Chile; 3 Escuela de Kinesiología, Facultad de Ciencias, Universidad Mayor, Santiago, Chile; 4 Laboratorio Integrativo Biomecánica y Fisiología del Ejercicio, Escuela de Kinesiología, Universidad de los Andes, Santiago, Chile; 5 School of Human Movement and Nutrition Sciences, The University of Queensland, Brisbane, Australia; 6 Facultad de Ciencias de la Rehabilitación, Universidad Andrés Bello, Santiago, Chile; 7 Universidad Pablo de Olavide ES-41013 Sevilla, España; 8 Universidad de Los Andes, Santiago, Chile; University of L'Aquila, ITALY

## Abstract

The sensorimotor system helps to maintain functional joint stability during movement. After anterior cruciate ligament (ACL) injury and reconstruction, several sensorimotor deficits may arise, including altered proprioception and changes in neuromuscular control. It is still unknown whether the type of autograft used in the reconstruction may influence knee sensorimotor impairments. The aim of this study was to comparatively assess the effects of the hamstring tendon (HT) and bone-patellar tendon-bone (BPTB) ACL reconstruction techniques on knee sensorimotor control 6–12 months post-operation. A total of 83 male subjects participated in this study: 27 healthy participants, 30 BPTB-operated patients and 26 HT-operated patients. Active joint position sense in 3 ranges of motion (90–60°, 60–30°, and 30–0° of knee flexion), isometric steadiness, and onset of muscle activation were used to compare sensorimotor system function between groups. Both operated groups had a small (< 5°) but significant joint position sense error in the 30–0° range when compared to the healthy group. No significant differences were found between the operated and the control groups for isometric steadiness or onset of muscle activation. The results of this study suggest that operated patients present knee proprioceptive deficits independently of surgical technique. Nevertheless, the clinical implications of this impairment are still unknown. It seems that selected surgical approach for ACL reconstruction do not affect functioning of the sensorimotor system to a large degree.

## Introduction

The sensorimotor system encompasses all the afferent, efferent, and central integration- processing components involved in maintaining functional joint stability during body movements [1, 2]. Inadequate functioning of this system may predispose joint damage [3, 4], a situation that commonly occurs after ligament injury. Following anterior cruciate ligament (ACL) injury, various sensorimotor impairments may occur, including proprioceptive deficit [5–10], decreased quadriceps and hamstrings strength [11–13], and alterations in muscle activation onset patterns [14]. Moreover, an ACL injury can produce alterations in ligament afferent signals that provoke altered brain activity patterns during knee movements [15]. While some deficits can be compensated by a greater flow of afferent information from other somatosensory pathways (e.g. visual feedback) [16], in some patients, these compensations are insufficient for recovering functional joint stability.

When the conservative treatment for ACL injury is unsuccessful in restoring knee joint functionality, ACL reconstruction (ACLR) may be indicated to restore joint stability. However, sensorimotor impairments and dynamic deficiencies may persist after surgery [17]. For instance, muscle recruitment patterns [18] and muscle strength [19] may remain altered in asymptomatic subjects even when some clinical indicators are within normal ranges. In an extensive literature review, Gokeler et al. [20] indicated that proprioceptive deficits detected by commonly used tests in ACLR patients are not conclusive in detecting sensorimotor impairments. As such, a comprehensive sensorimotor evaluation is needed to assess the function of the joint before returning to normal activities, a decision that in some cases, is based on subjective judgement or time-after-surgery criteria [21]. Assessments that evaluate the integrity and function of sensorimotor components by measuring variables along the afferent or efferent pathways may be used to obtain broader information about joint functionality after ACL injury and surgery [22].

In ACLR patients, reconstruction type may be an important factor related to impaired sensorimotor control [23]. For instance, during the post-operative stage, the graft used for the reconstruction contributes to tissue and mechanoreceptors regeneration [24, 25]. Nevertheless, the exact time needed for mechanoreceptors to appear is unclear, as is the time needed for other somatosensory pathways to compensate for the lower afferent signals coming from the ligament [25, 26]. This altered afferent information results in knee sensorimotor deficits, which may be dependent on the autograft used for ACLR [27]. While quadriceps muscle strength/activation deficits [28], knee proprioception impairment [8], and altered muscle activation patterns [29–31], among other alterations, are present in patients who receive either a bone patellar tendon bone (BPTB) graft or a hamstring tendon (HT) graft, some studies report differences in sensorimotor impairments between graft types [32, 33]. Therefore, existing knowledge on the effects of graft type on sensorimotor control of the knee is still conflicting.

The aim of this study was to comparatively assess the effects of the HT and BPTB ACL reconstruction techniques on knee sensorimotor control in patients 6–12 months post-operation. Essential methods to assess the components of the sensorimotor system [22] were used including: joint-position sense (JPS), muscle tension sense (steadiness), and onset time of electromyographic (EMG) activity of knee muscles during an unexpected perturbation. These methods evaluate the afferent and efferent components of functional joint stability [22] that may be affected differently in each type of graft. Therefore, this information may help to establish the status of individual sensorimotor components that contributes to joint stability during the post-operative period and recovery phase [22]. Due to the nature of the surgery, we hypothesized that the type of graft would differentially impact at least one dimension of sensorimotor control.

## Materials and methods

### Participants

Participants were recruited by public announcements within the clinic and by contacting ACLR patients listed in the clinic’s database. A total of 30 healthy participants and 89 ACLR patients were contacted within a timeframe of 18 months and were interviewed to assess clinical eligibility. Subject exclusion criteria included the following: any previous surgical intervention in the lower extremities (healthy subjects); acute or chronic pain in the lower extremities within the past six months (healthy subjects); more than one concomitant injury/repair during surgery (e.g. tibial collateral ligament injury plus meniscus repair; operated subjects); body mass index higher than 30 (both groups); chronic ankle instability (both groups). After checking for inclusion and exclusion criteria, 83 males were invited to participate in the study, and were divided into three groups: control (n = 27), BPTB (n = 30) and HT (n = 26). Anthropometric characteristics of each group are presented in Table 1. All operated patients were assessed 6 to 12 months post-surgery (8.6 ± 2.3 months). All study participants scored between four and seven on the Tegner scale for physical activity [34].

**Table 1:** Anthropometric characteristics, time post-surgery and activity level of each group.

	Groups		
	Control (n = 27)	BPTB (n = 30)	HT (n = 26)
Age (years)	24.27 ± 3.28	25.77 ± 4.47	26.60 ± 5.74
Weight (kg)	75.90 ± 8.53	73.16 ± 7.46	78.08 ± 9.40
Height (m)	1.76 ± 0.06	1.72 ± 0.06	1.74 ± 0.06
BMI (kg/m^2^)	24.28 ± 2.07	24.69 ± 1.80	25.48 ± 2.42
Time Post-Surgery (months)	—	7.77 ± 2.28	8.92 ± 2.30
Activity Level (Tegner Scale)	6.40 ± 1.71	5.88 ± 1.41	5.39 ± 1.99

Characteristics of each study group. Values are mean ± standard deviation. HT: hamstring tendon; BPTB: bone-tendon-bone.

All reconstructed patients were rehabilitated at the same clinic with the same general aims for each rehabilitation phase [17]. However, specific precautions were taken for each type of graft, with excessive stress avoided over the anatomical zone where the graft was obtained. The aims of the first six weeks of rehabilitation were to reduce inflammation, restore the knee range of motion, favor muscle activation, and gait retraining. In the second stage, (six weeks post-surgery), the aims were to strengthen the knee musculature and increase neuromuscular control. In the third stage (12 weeks post-surgery), the aims were to normalize knee musculature strength and to start jogging. In the final stage (20 and 32 weeks post-surgery), the aim was to restore functional performance (e.g. in plyometric exercises, jumping, and changes in direction) [17]. All participants provided signed informed consent before participating in the study, which was approved by the Ethical Committee of the Faculty of Medicine, Pontificia Universidad Catolica de Chile and was conducted in accordance with the Declaration of Helsinki.

### Measurements

The testing order was randomized by the investigators. Grouping assignments were sorted by the principal investigator, who was blinded to information regarding surgical method and the post-operation time of each patient. The sensorimotor control of the knee was measured through: (a) JPS in three distinct ranges of joint movement; (b) quadriceps muscle tension sensation using the isometric steadiness technique, and; (c) EMG onset of muscle activation of vastus medialis, vastus lateralis, semitendinosus, and biceps femoris muscles after an unexpected perturbation. The test side for ACLR subjects was the reconstructed leg, whereas the test side for control subjects was pseudorandomly selected to counterbalance the ACLR limbs. This resulted in three groups that were used for comparisons: (i) leg of the healthy subjects (control group); (ii) reconstructed leg of patients operated with the HT method; and (iii) reconstructed leg of patients operated with the BPTB method.

#### Joint-position sense

**Fig 1:**
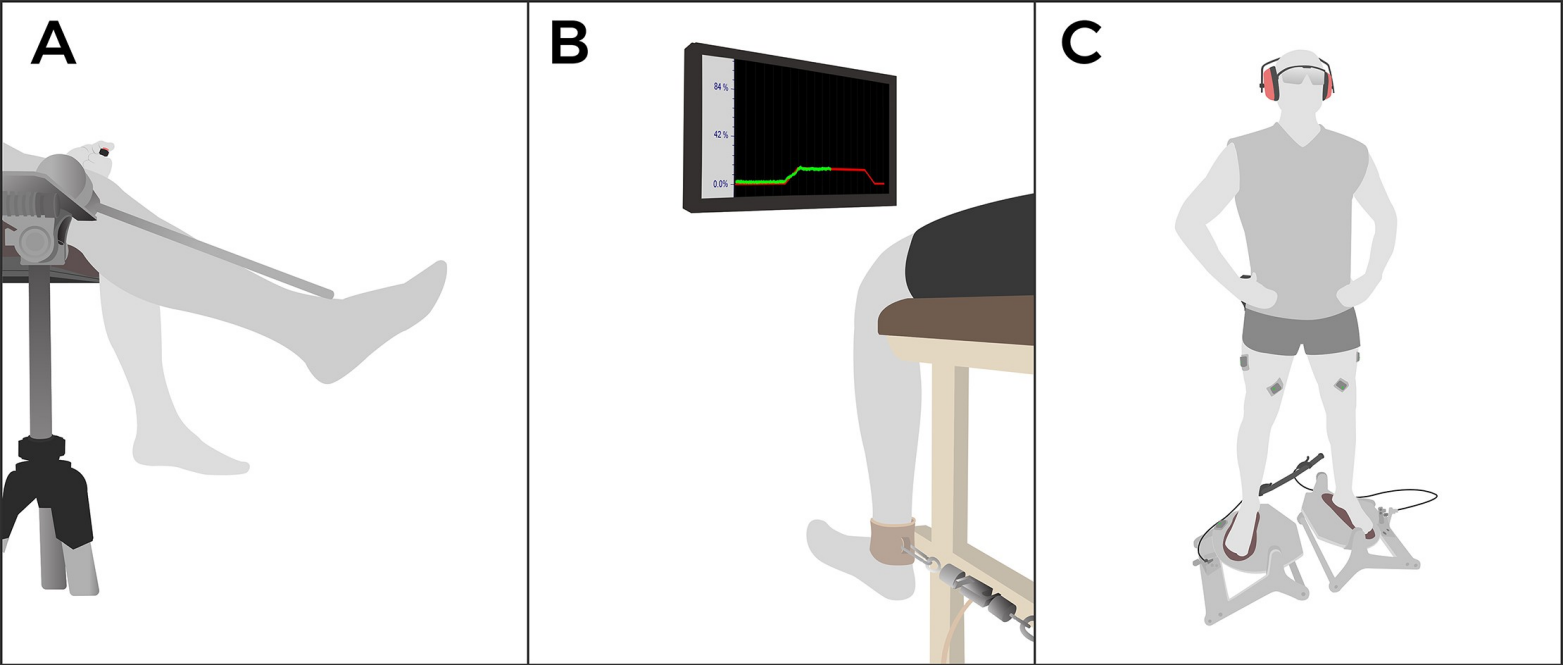
Knee sensorimotor assessment. **A** Joint-position sense test with the uniaxial electrogoniometer. **B** Steadiness test (green line) with the load cell and paradigm set to 15% maximum isometric voluntary contraction (red line). **C** Muscle activation onset test in the knee, showing the positions of the surface EMG sensors and platform.

The aim of this test was to evaluate JPS, the ability of subjects to actively replicate a previously determined joint position [22, 35]. Angular measurements of the knee joint were taken with a uniaxial electrogoniometer (Kinetecnics, Santiago, Chile) using an incremental encoder (Hengstler, Aldingen, Germany) connected to a computer. The encoder was adapted for the patient with a rod, enabling knee angle assessment (Fig 1A). This device was shown to have a good resolution (5000 pulses, 0.072° of resolution) with a high reproducibility rate (intraclass correlation coefficient = 0.999). As previously reported [36], three ranges of movement (90–60°, 60–30°, and 30–0°) were used [5, 7], with 0° being knee full extension. These ranges were chosen because previous studies suggest that afferent discharge of capsule-ligamentous proprioceptors is influenced by joint angle, where different discharge rates occur within the knee range of motion [37]

For the test, each subject was seated on the edge of a stretcher, with the fulcrum of the electrogoniometer aligned with the axis of the knee (i.e. lateral femoral condyle). For each range, a target knee angle was first achieved using visual feedback on a computer screen to ensure that the subject selected the target within the ranges of movement pre-established for the evaluation. While the subject was extending their knee, a green light appeared on the screen if the knee angle was within the range of movement, and a red light appeared if the knee angle was out of range. Once a target angle was chosen, the subject pushed a button associated with the encoder system, which saved the selected target angle in the computer. In other words, each subject chose any angle within the range of movement as the target angle for the JPS assessment, angle that was used in the consecutive repetitions. This procedure was repeated for each of the assessed ranges (i.e., 90–60°, 60–30°, and 30–0°), obtaining three different target angles for each of the ranges [36]. Later, without visual feedback, the subject tried to reproduce the target angle for each of the ranges. Independent of the evaluated range of movement, subjects started from 90° of knee flexion towards extension. This procedure was repeated 10 times for each extremity and for each of the defined ranges of joint movement. Therefore, a total of 30 repetitions were obtained. For each repetition, the absolute value of the difference between the achieved angle and the target angle was calculated (e.g. error angle). Then, the differences of the 10 repetitions were finally averaged for each range. The joint position signals from the electrogoniometer were processed on Igor Pro 6.0 software (WaveMetrics Inc, Lake Oswego, USA).

#### Muscle tension sensation: Steadiness

The purpose of this assessment was to evaluate the ability of subjects to maintain a constant force at 15% of maximum voluntary isometric contraction, which reflects fine muscle control [38, 39]. Isometric steadiness was represented as the coefficient of variation between the target and the force maintained by the subject. First, the maximum voluntary isometric contraction (MVIC) was assessed. Participants were seated in an instrumented chair with approximately 90° of knee flexion, with a load cell (Scottdale, Arizona, USA) anchored to the distal end of the leg at the level of the ankle (Fig 1B). Participants were asked to exert an MVIC of the knee extensor muscles for 4 s. This procedure was repeated three times, with a rest period of 1 min between each repetition. The signal from the load cell was captured with a Trigno Wireless System amplifier (Delsys, Boston, USA) at a sample frequency of 2000 Hz, where the maximum force obtained between the three repetitions was selected for further analysis.

Knee isometric steadiness was evaluated with the same setup as the MVIC assessment. A computer screen projected a trapezoidal figure (Fig 1B), where the upper part represented the 15% of the previously measured MVIC. Each subject was asked to exert knee extensor force to reach this target, coinciding with the projected trapezoidal figure. Real-time feedback on the exerted force was provided so that the task could be sustained for 20 s and to match the trapezoidal target. Subjects performed four practice trials separated by 45 seconds of rest. After practice, subjects rested for two minutes and then performed three repetitions of the task, with a rest period of 1 min between each repetition. Isometric steadiness was quantified as the coefficient of variation between isometric strength fluctuations around the projected trapezoidal target [38, 39]. Stabilization of the exerted force occurs normally during the first 8 seconds of the contraction [40], therefore, a visual criteria was used to select the start of the stable signal. A 10-second window of analysis was used thereafter, beginning from the selected start of the stable signal. This method has been previously used to analyze the most stable part of the generated force and has been proven reliable [39, 41]. The final obtained result was the coefficient of variation (%) of the three repetitions, which is a measure of statistical dispersion that describes the degree of variability between measurements. Igor Pro 6.0 software was used to calculate isometric steadiness.

#### Onset time of muscle activation in the knee muscles

The onset of muscle activation in the knee muscles was estimated utilizing surface electromyography. EMG bipolar sensors (Delsys, Boston, USA) were positioned on the vastus medialis, vastus lateralis, semitendinosus, and biceps femoris muscles of each subject according to SENIAM recommendations [42]. EMG signals were pre-amplified in a simple differential manner, filtered in a bandwidth of 50–450 Hz, and recorded at a sampling frequency of 2000 Hz (Trigno Wireless System, Delsys, Boston, USA). Two destabilizing platforms elicited perturbations in both lower limbs as previously reported [22, 30, 43] (Fig 1C). A sudden fall of the platforms causes 20° of inversion at the ankle in a weight-bearing condition. This mechanical perturbation over the ankle causes a generalized lower limb destabilization, inducing neuromuscular responses to overcome a loss in balance [35]. The aim of this method is to stimulate the lower limb in a position that ensures muscle activation, joint capsule compression, and stretching of the skin during the evaluation, all of which is crucial information for central proprioception and balance control [35, 44].

The fall of the platform was detected with a triaxial accelerometer (Delsys, Boston, USA) that was synchronized with the EMG signals. This procedure was performed six times for each extremity, with the side of perturbation being randomly selected. Blindfolds and earplugs were used with each participant to nullify the effects of vision and hearing in the evaluations. To calculate the onset of EMG activity, the signals were fully rectified using the Average Rectified Value (AVR) method. Then, an activation threshold was established as the average of the basal amplitude plus five standard deviations in a window of 500 ms measured 50 ms prior to the perturbation. Thus, the start of the muscle activation was defined as any EMG burst that exceeds the activation threshold [45, 46]. A computational script (Igor Pro 6.0, WaveMetrics Inc, Lake Oswego, USA) plus visual inspection allowed for the semiautomatic detection of muscle onset time, defined as the time between the start of perturbation (i.e., stop after the fall of the platform) and the start of EMG muscle activation. This procedure was performed for each of the muscles evaluated. Three randomly selected repetitions were averaged, obtaining the EMG onset latency, which was considered for further analysis.

### Statistical analysis

Sample size was estimated based on a one-way analysis of variance (ANOVA) design. Using an alpha level of 0.05 adjusted by multiple comparison tests after ANOVA, a statistical power of 0.9 and an estimated effect size of at least 0.8 (Hedges´g) on the difference of average angle for joint reposition, a sample size of 10 participants per group was obtained [5]. The Shapiro-Wilk test was used to evaluate normality assumptions for all continuous variables. Thus, according to data distribution, a one-way analysis of variance (ANOVA) test or a Kruskal-Wallis test was used to compare JPS, steadiness, and EMG onset among study groups (i.e.: Control, HT, BPTB). A multiple comparison post hoc test (Scheffé or Dunn’s) was used after ANOVA. All statistical analyses were performed using the STATA 9.1 software. The level of significance was set at α = 0.05.

## Results

### Joint-position sense

**Fig 2:**
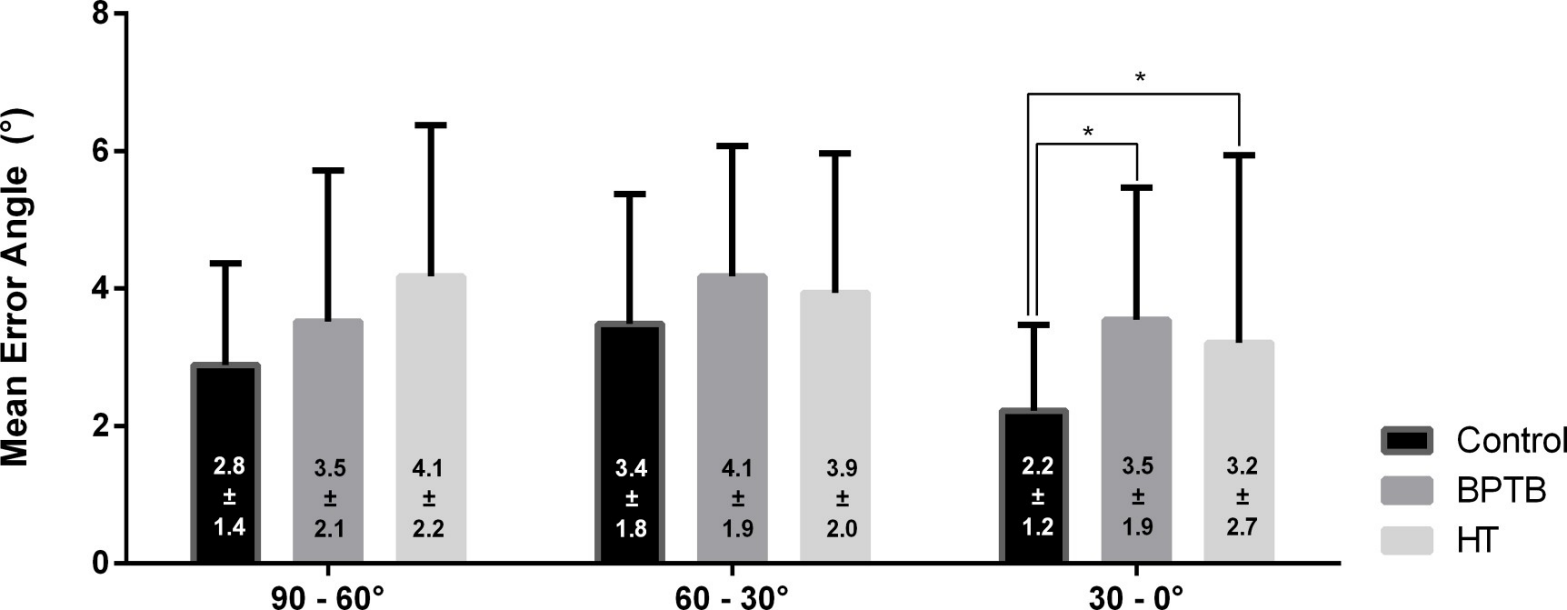
Joint position sense results. Comparative results for JPS between the control, HT, and BPTB groups for the three different angle ranges. Bars represent the mean ± standard deviation (error bars). * indicates significant differences (p < 0.05) between the assessed variables. Abbreviations: JPS, joint-position sense; HT, hamstrings tendon; BPTB, bone-patellar tendon-bone.

The BPTB and HT groups showed significantly higher error values in comparison to the control group in the 30–0° range (p = 0.017 and p = 0.039 respectively) (Fig 2). No other significant differences were found when comparing JPS results between control, BPTB, and HT groups. Details of the JPS results are provided in Fig 2.

### Muscle tension sense: Steadiness

**Fig 3:**
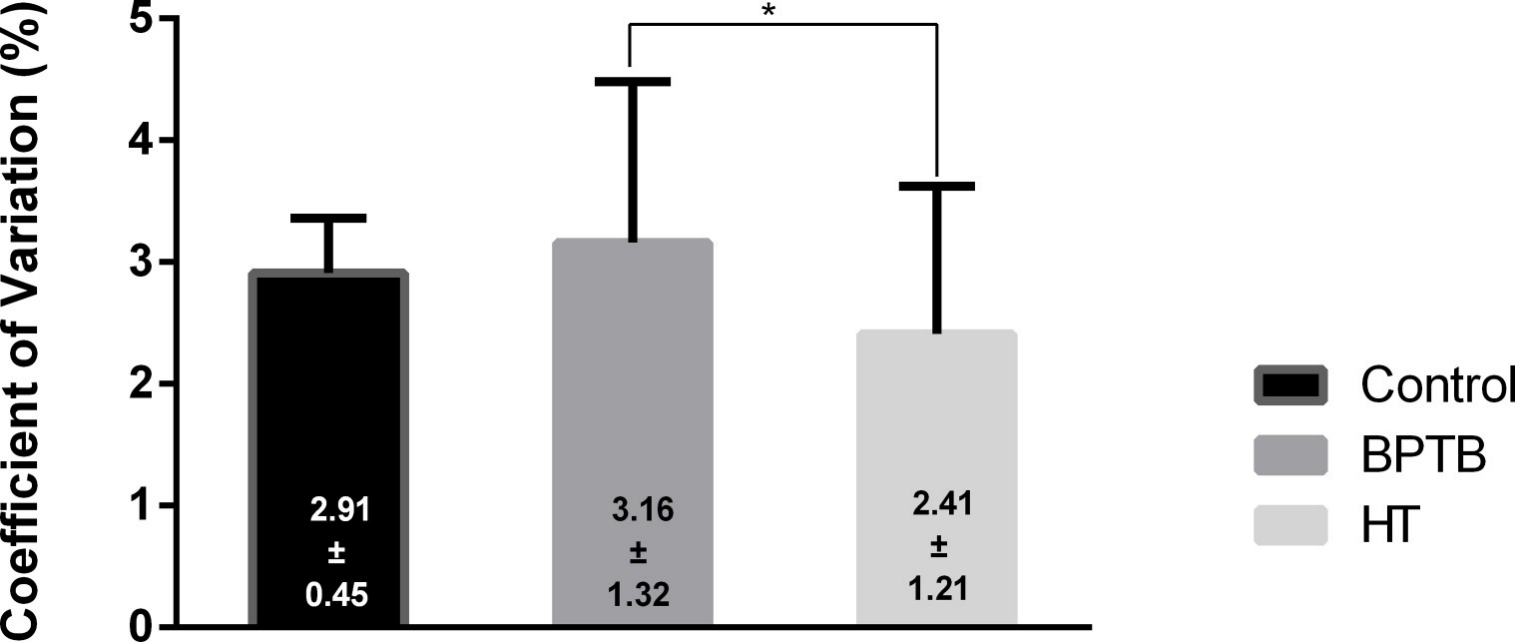
Isometric steadiness results. Comparative results for isometric steadiness between the control, HT, and BPTB groups. Bars represent the mean ± standard deviation (error bars). Abbreviations: HT, hamstrings tendon; BPTB, bone-patellar tendon-bone.

The coefficient of variation was 2.91 ± 0.45% for the control, 3.16 ± 1.32% for the BPTB, and 2.41 ± 1.21% for the HT groups. A significant difference was found between the HT and BPTB groups (p = 0.041) but no difference was found between the HT (p = 0.061) and BPTB (p = 0.99) vs the control group (Fig 3).

### Onset of muscle activation in the knee

No significant differences were found when comparing the onset of muscle activation for the vastus medialis, vastus lateralis, semitendinosus, and biceps femoris muscles between the Control, BPTB, and HT groups. Details of these results are presented in Table 2.

**Table 2:** Onset of muscle activation for the control and operated groups.

	Vastus medialis (ms)	Vastus lateralis (ms)	Semitendinosus (ms)	Biceps femoris (ms)
Control	97.54 ± 11.11	99.68 ± 13.70	96.85 ± 8.97	101.2 ± 9.35
BPTB	91.48 ± 11.01	90.95 ± 12.17	91.44 ± 12.12	98.75 ± 11.03
HT	93.41 ± 16.19	93.90 ± 17.29	91.38 ± 12.98	94.00 ± 13.66

Onset of muscle activation for the different muscles and groups. Values are mean ± standard deviation. HT: hamstring tendon; BPTB: bone-tendon-bone.

## Discussion

The aim of this study was to determine differences in knee sensorimotor control of ACLR patients operated with HT and BPTB techniques. Our results indicate that 6 to 12 months after surgery, steadiness and onset of muscle activation are similar between both operated groups and the control group. However, near full knee extension, small magnitude JPS errors can persist in operated patients independently of surgical approach. While this detriment in JPS is significant, the clinical implications of this finding remain unknown.

### Joint-position sense

Both operated groups tended to present a higher JPS error than the control group near knee full extension. Previous evidence support these findings in athletic [9] and non-athletic populations [10]. Increased errors in JPS in the reconstructed groups may be explained by two theories. Firstly, an increased error in the capacity to reproduce specific joint positions may be interpreted as an alteration in the modulation of proprioceptive information by the muscle spindle [35, 37]. It has been proposed that the muscle spindle is the final common input of sensory information that the central nervous system uses as feedback for joint position [47] and sensorimotor control. That is, most of the proprioceptive information provided by joint receptors go through the same afferent pathway of the muscle spindle. The spindle contributes to muscle force control and joint position sense through a spinal circuit known as the gamma (γ) loop. This circuit is formed by γ-motoneurons that transmit excitatory/inhibitory pulses to α-motoneurons via Ia afferents, reflex loop that can be modulated by afferent information [48]. Since mechanoreceptors in the ACL provide important afferent information on the relative position and movement of the knee joint [1, 9], ACL injury and reconstruction appears to impair proprioceptive ability through disruption of the transmission and modulation of this sensory information. Abnormal neurologic output from the articular capsule, the collateral and the posterior cruciate ligaments may also contribute to this abnormal spindle modulation. Furthermore, capsuloligamentous structures seems to be more sensitive to joint position changes near knee extension [37], which may be related with our findings of altered JPS just in the 30–0° range. Therefore, the alteration of JPS in the operated subjects may indicate a reduced joint position sense ability due to disruption in muscle spindles’ modulation of proprioceptive information.

Another possible explanation for the altered JPS may be related with changes in central nervous system (CNS) after injury and reconstruction. It has been demonstrated that ACL reconstruction and subsequent rehabilitation process evoke neuroplastic changes at the level motor cortex that are not normalized after treatment or return to activity [49]. Indeed, these modifications may elicit supraspinal inhibition of voluntary muscle activation [50], and/or altered neuromuscular control during functional tasks [51]. Even simple cognitive and sensorimotor tasks present higher attentional demands [52] and neurocognitive overload [51] in this kind of patients. This supports the concept that somatosensory, neuroplastic, cognitive, and visual-motor changes can occur after ACL injury and reconstruction [15]. Therefore, the JPS errors in this study may result from CNS reorganization and subsequent altered processing of sensory information, which according to our results, seems to be similar for the HT and BPTB surgical techniques. Our finding of altered JPS in the operated groups can be interpreted from a different point of view. While significant, the recorded error values for JPS were small (~3°) and fall below values established for the clinical relevance (5°) of similar techniques [53]. Furthermore, it seems that there is no substantial evidence of a strong relationship between joint position sense ability and functional performance [20]. Previous studies using H-reflexes to evaluate neuromuscular control in ACLR patients, have found that muscle spindle function is restored within the first 6 months post-surgery [28]. Further, it has been suggested an increased neuromuscular excitability (increased H-reflex amplitude) in patients receiving HT grafts [54]. Therefore, our JPS findings could be considered circumstantial. However, JPS and H-reflex evaluations when used to infer muscle spindle function are methodologically different, thus the obtained results may be interpreted differently. For instance, it can be argued that joint repositioning includes a higher proportion of descending activation/inhibition from supraspinal centers [1, 35], in comparison to H-reflexes which are considered in essence an estimation of spinal excitability [55]. Overall, the results of this study interpreted in light of the current literature suggest that the altered JPS found in the operated patients may not have clinical implications.

### Muscle tension sense: Steadiness

Force steadiness was not altered in the HT or BPTP groups in comparison to the controls. To our knowledge, this is the first study comparing this variable between patients with different ACL surgical grafts. Force fluctuation is dependent on the interaction of multiple features of motor unit behavior, which change as a function of contraction intensity [56]. Alterations in motor unit recruitment and rate coding properties or adaptations in the activation pattern of the motor unit population (e.g., motor unit synchronization and coherence) would affect force variability [57]. Impairments in force steadiness have been associated with ACL injury and ACLR [12]. Considering this, the obtained steadiness results suggest that, by 6–12 months post-surgery, both assessed patient groups (HT and BPTP) had adequately adapted neuromotor control of the knee muscles and were, therefore, able to reduce force fluctuations during isometric contractions. While the exact mechanisms of this adaptation are unknown, several hypotheses can be postulated. First, the post-operative period (i.e., 6–12 months) may have been enough for muscle contraction to recover. Although ACL injury and the degree of graft regeneration are linked to muscle weakness [16, 28], quadriceps and hamstrings strength recovers early during the rehabilitation period after ACLR [58] regardless of the degree of graft regeneration [23, 59] probably due to neuromuscular adaptations such as enhanced ligament-muscular reflex arc excitability [60]. Second, neuromuscular adaptations such as increased antagonist coactivation may increase fine muscle control and force steadiness, thus increasing muscle stiffness and joint stability [11, 12]. Moreover, lower limb training such as within the rehabilitation process, may improve force steadiness directly [61] or indirectly through increased muscle coactivation [62]. Therefore, the results of the present study suggest that commonly used rehabilitation protocols (as used with the assessed patient groups [17]) may restore force steadiness 6 to 12 months after ACL surgery, independent of the type of graft used in the surgery. Future studies may use prospective designs to clarify the effect of the rehabilitation protocols in force steadiness.

### Muscle onset timing after ACLR

The patients in both operated groups presented similar muscle onset values at the moment of evaluation. In humans, long latency muscle stretch reflex responses occur within 50–200 ms following an external stimulus [63]. This protective reflex is a rapid muscle response that keeps the joint stable against perturbations that put stability at risk [64]. Deficits in sensory information could alter the latency of a reflex response, thus increasing the risk for joint instability [1]. Prior research has reported that the onset of muscle activation can be delayed in patients with joint instability [52] and ACLR [63]. For instance, a recent study found that ACL reconstructed knees present altered muscle onset timing after ankle perturbations while standing [30]. The possible cause of these differences may be related with the magnitude of the destabilization (30° of inversion, 10° plantarflexion in their study compared to 20° of inversion in this study). However, our comprehensive rehabilitation process over a large sample size make us believe that the onset of muscle activation, and the other components of sensorimotor control evaluated here, may have improved 6 to 12 months post-surgery. Previous literature supports neuroplastic changes following simple exercise, balance and resistive training, and motor task training [49]. These interventions may enhance neurogenesis, improve cognitive function, and modify nervous system excitability [49], thus enhancing neuromuscular control after ACL reconstruction. Indeed, supraspinal reorganization may contribute to the restoration of long latency muscle stretch reflex responses [60], compensating for possible deficits in afferent information from the joint. Specific muscle strengthening [18] and sensorimotor training can also increase knee joint stability via enhanced reflex excitability and/or an earlier recruitment of motoneurons [65]. Thus, the rehabilitation process that the ACLR group of this study underwent may have partly reestablishing normal functioning of the sensorimotor system after changes produced by injury and reconstruction. This may be related with the lack of differences found between ACL reconstructed groups and the controls. However, these assumptions remain as speculations due to the cross-sectional nature of our cohort. Prospective studies are needed to shed light on the effects of ACLR rehabilitation protocols on specific components of the sensorimotor system.

### Study limitations and future research

The results of this study should be interpreted considering some limitations. As mentioned before, the JPS difference found between the control and HT groups was small and the clinical importance of this change remains undetermined. Since afferent information to the muscle spindle may be influenced in part by joint position, future studies should consider more sensitive methods to asses JPS and spindle function such as the H-reflex [66]. Regarding steadiness, the measurement of this variable at different joint positions might be desirable, since knee position may have an effect on muscle force production and muscle activation [67]. In relation with the measurement of muscle onset around the knee, ankle perturbations may be considered non-specific as compared to other methods proposed in the literature [63, 68]. Furthermore, the magnitude of the perturbation might not be enough to elicit a clear response in the knee muscles [30]. However, distal perturbations in a weight bearing situation includes the proprioceptive information of the ankle, which is crucial in the assessment of lower limb sensorimotor control [35] and might be important in the modulation of muscle spindle function. Therefore, we believe that this method contributes to assessing the efferent pathways of the sensorimotor system related to knee stability [22]. Whereas our preliminary results included women’s in our sample, we were unable to have similar proportions of males and females between ACLR groups. As it there is a possibility that the male response may drive the differences with no effect in females, or there can be interactions between gender and the dependent variables, we opted to exclude women from the final analysis. This means that our conclusions can only be drawn for male patients. Finally, since sensorimotor impairments after ACLR have been found at the motor cortex [16] and spine [54], future studies should assess these levels of the sensorimotor system. Measurement techniques such as functional MRI, transcranial magnetic stimulation and H-reflexes among others may be needed to explore central nervous system reorganization after ACLR.

## Conclusions

ACLR individuals have an altered JPS near knee extension (30–0°) when compared to control subjects, independently of the surgical approach utilized. This suggest altered modulation and/or processing of proprioceptive information. Because JPS impairment was small in magnitude (~3° of error), and since no other altered sensorimotor components (e.g., force steadiness, muscle onset) were found, the impact of this finding on knee function is arguable. Therefore, the surgical approach used for ACLR appears to not affect the sensorimotor behavior of the knee 6 to 12 months after surgery.
